# Mixed-Reality Visualization of Impacted Teeth: A Survey of Undergraduate Dental Students

**DOI:** 10.3390/jcm14196930

**Published:** 2025-09-30

**Authors:** Agnieszka Garlicka, Małgorzata Bilińska, Karolina Kramarczyk, Kuba Chrobociński, Przemysław Korzeniowski, Piotr S. Fudalej

**Affiliations:** 1 Department of Orthodontics, Jagiellonian University in Kraków, 31-155 Kraków, Poland; 2 Department of Oral Surgery, Jagiellonian University in Kraków, 31-155 Kraków, Poland; 3 Sano Centre for Computational Medicine, 30-054 Kraków, Poland; 4 Department of Orthodontics and Dentofacial Orthopedics, School of Dental Medicine/Medical Faculty, University of Bern, 3010 Bern, Switzerland

**Keywords:** mixed reality, impacted tooth, dental education

## Abstract

**Background/Objectives**: Integrating 3D visualization technologies, including virtual reality (VR), augmented reality (AR), and mixed reality (MR), into dental education may enhance students’ understanding of facial anatomy and clinical procedures. This study aimed to assess dental students’ perceptions of using MR for three-dimensional visualizations of impacted teeth. **Methods**: Cone-beam computed tomography (CBCT) scans of patients with impacted teeth were retrospectively selected from a university clinic database. The CBCT images were processed to adjust contrast for optimal visualization before being uploaded to MR goggles (HoloLens 2). A total of 114 final-year dental students participated, each manipulating the 3D images in space using the goggles. Following this, they completed a seven-question survey on a five-point Likert scale (1 = strongly agree, 5 = strongly disagree), evaluating image quality and the usefulness of 3D visualization. **Results**: The study group consisted of 29 males and 85 females (mean age = 24.11 years, SD = 1.48). The most favorable responses were for enhanced visualization of the impacted tooth’s position relative to adjacent structures and the inclusion of 3D image visualization as a teaching aid, which benefited students while learning and allowed them to better understand the course of the procedure for exposure/extraction of the impacted tooth, with median scores of 1, indicating a highly favorable opinion. A statistically significant relationship was found between the responses of females and males regarding the quality of the presented image using HoloLens 2 goggles. No significant correlation was found between participants with and without prior experience using VR/MR/AR. No significant correlation was found between age and responses. **Conclusions**: Students reported an improved understanding of the relationships between impacted teeth and adjacent structures, as well as potential benefits for clinical training. These findings demonstrate a high level of acceptance of MR technology among students; however, further research is required to objectively assess its effectiveness in enhancing learning outcomes.

## 1. Introduction

Continuous progress in digital technology forces us to look for new solutions to upgrade the learning process. Augmented-reality (AR), mixed-reality (MR), and virtual-reality (VR) modalities provide new directions for visual representation and the transfer of knowledge through images [1]. A better presentation of scientific concepts can enhance learning effectiveness. Finding the three-dimensional (3D) relationship of the structures, manipulating the images, and creating records are a few examples of the huge potential of the usage of 3D imaging [2]. However, when starting to work with new technology, we must familiarize ourselves with the advantages and possibilities it brings and be aware of its disadvantages [3].

Introducing 3D visualization technology of facial anatomical structures into common use in academic education may be a promising tool for students during teaching classes.

Existing studies suggest that 3D visualization can be a promising educational tool during university and beyond. The research used both printed 3D models and VR, AR, and MR technologies, and the appropriately prepared surveys were used to assess satisfaction with the presented technology. The conclusions that were drawn from the research are that MR offers a realistic three-dimensional perspective of the face, enhancing anatomy education through a more captivating approach [1] and VR interfaces enhance comprehension of craniofacial trauma and enable accurate depiction of craniofacial anatomy [4]. Additionally, the three-dimensional visualization technology allowed for a certain type of training related to performing specialized activities in the future, which boosted the confidence of dental students, demonstrated an improved learning experience and developed is suitable for evaluation with surgical trainees [5,6,7].

Our study aims to conduct a survey in which we will collect the opinions of dental students to find out how they evaluate visualizations using three-dimensional technology, especially in the context of cone beam computed tomography (CBCT) images of impacted teeth. Impacted and displaced teeth are teeth that are unable to erupt due to obstruction by other teeth or bone or are positioned abnormally [8]. This problem is relatively common affecting up to 20% of the population [9]. Future dentists should understand this condition, diagnose it quickly, and know when to refer the patient to an orthodontist. By collecting and analyzing these opinions, we intend to provide reliable data on the acceptance and potential benefits of introducing 3D visualization technology into education at medical universities.

## 2. Materials and Methods

All procedures related to the study were conducted on anonymized files in full compliance with the Declaration of Helsinki and Good Clinical Practice (GCP) guidelines. This study received ethical approval from the research ethics committee at the Jagiellonian University Medical College (expertise number 118.0043.1.73.2024).

### 2.1. Acquisition and Preparation of 3D Images

**Figure 1:**
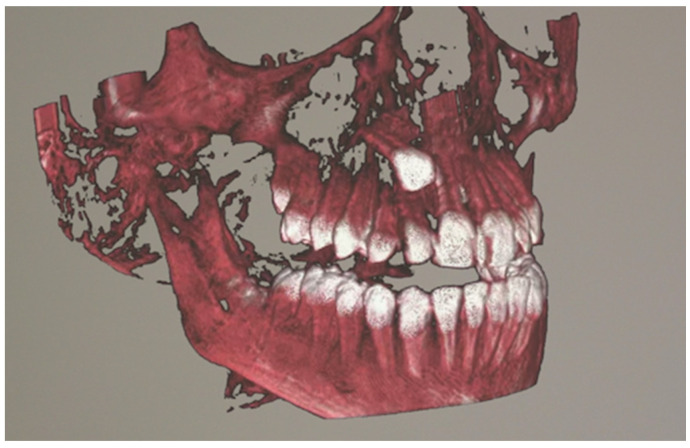
Visualization of an impacted canine in mixed-reality using HoloLens 2, right lateral view.

**Figure 2:**
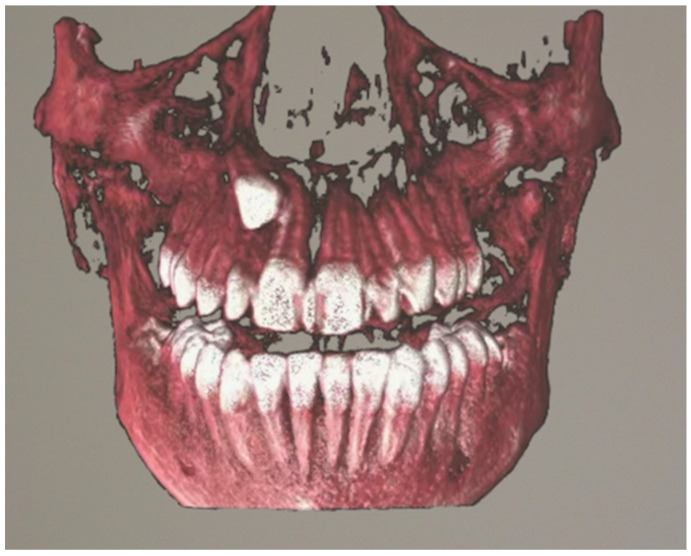
Visualization of an impacted canine in mixed-reality using HoloLens 2, frontal view.

Patients with impacted teeth undergoing CBCT were identified from the University Dental Clinic in Kraków data. All qualified tomography scans were approved by an orthodontic specialist so that they presented an image corresponding to the most common images of impacted teeth. The selected data was exported in the Digital Imaging and Communications in Medicine (DICOM) file format. 10 radiographs of selected studies included cases of impacted maxillary canine, an impacted mandibular canine, an impacted mandibular molar, an impacted incisor, and multiple impacted teeth. The average age of the patient whose tomography was visualized using the headset was 12.4 years. The CBCT scans were acquired using a KaVo OP 3D PRO unit (Kavo Dental, Biberach, Germany) with the following parameters: 90 kV, 5 mAs, 8 ms. The field of view (FOV) was identical for all radiographic examinations analyzed, with a parameter of 78 × 150 mm. CBCT radiographs were anonymized, and then subjected to semi-automatic segmentation. The segmented CBCT was uploaded to an application that allows visualization of three-dimensional images using HoloLens 2 goggles (Microsoft, Redmond, WA, USA) [10]. Figure 1 and Figure 2 present the visualization of impacted canine in mixed reality.

The HoloLens2 goggles proposed in the study, using the so-called mixed-reality (MR). MR combines elements of both the digital world and the real world. The software used for this study was developed using Unity Game Engine specifically for this use case, including functionalities for exploration and interaction with the presented data. Three-dimensional images are displayed and anchored/localized using goggles in the real world. This allows the user to interact with the image, using hand gestures to perform various maneuvers such as enlarging, reducing, or rotating the image [11].

### 2.2. Method Evaluation

Fifth-year medical and dental students were qualified for the study, since they had already completed classes on impacted teeth during classes in dental surgery and orthodontics. The inclusion criterion was admission to orthodontic classes. The exclusion criteria included the use of corrective glasses that prevented the correct application of HoloLens 2 goggles, failure to consent to participate in the study, epilepsy, sensitivity to light stimuli, and a pacemaker. After meeting the criteria, 114 students were included in the study. A priori, the study participants received an information sheet and an explanation of MR technology. They were given the opportunity to ask questions. Before the practical session, the students attended a PowerPoint presentation that summarized the key information regarding impacted teeth and provided a brief explanation of VR/AR/MR technologies. Following the presentation, each participant underwent a two-minute individual training session on the use of the HoloLens 2 goggles, which included instructions on image manipulation and device operation. All students were trained by the same instructor, and a single investigator was responsible for collecting all study data. If needed, the HoloLens2 was calibrated for the participant. The previously uploaded radiographs of impacted teeth were presented to them.

Participants were asked to complete an anonymous survey assessing satisfaction with the presented image. The survey consisted of 7 questions (Table 1):

1. The three-dimensional visualization helped you evaluate the position of the impacted tooth.
2. You did not have any problems with the image manipulation using the HoloLens 2 goggles.
3. The quality of the presented image using HoloLens 2 goggles is satisfactory.
4. Would you like three-dimensional image visualization to be an additional element of teaching classes?
5. The three-dimensional representation of the image will benefit you while learning.
6. The three-dimensional visualization of the impacted tooth allowed you to better understand the course of the procedure of exposure/extraction of the impacted tooth.
7. The three-dimensional image of the impacted tooth allowed you to better understand the possible complications associated with the treatment of impacted teeth.

Responses were gathered using a 5-point on a scale from 5 = strongly disagree to 1 = strongly agree. Answers such as “I don’t know” were not recorded and were not allowed.

All questions were developed by academic teachers with a minimum of three years of experience in teaching dental students. Subsequently, the questions were reviewed by 10 practicing dentists to assess whether they were written in a clear and comprehensible manner.

### 2.3. Statistical Analysis

Descriptive statistics, such as mean and standard deviation of age, were used to describe the population. Student evaluations of the use of HoloLens goggles were collected using a Likert scale. The responses were treated as ordinal variables for statistical analysis. Owing to the non-normal distribution of the data, descriptive statistics were reported using the median and quartiles rather than the mean and standard deviation. Continuous data was compared with a Mann–Whitney test, as all the variables had non-normal variables. All the tests were unpaired and two-sided with a confidence interval of 95%.

All the analyses were performed with IBM SPSS Statistics, Version 29.0.2.0 (20).

## 3. Results

From all eligible samples 139 students, a total of 114 students (82%) took part in the presented study. Mean age was 24.11 (SD = 1.48). The youngest participant was 22 years old and the oldest was 30. There were 29 men and 85 women. None of the participants withdrew while presenting the radiographs. Among the study participants, 85 individuals reported prior experience with VR/MR/AR headset technology, 15 indicated no such experience, and one respondent did not answer the question. 

**Figure 3:**
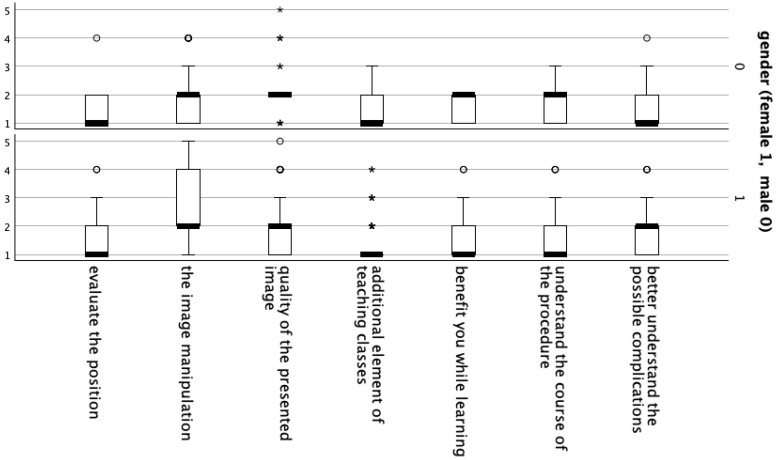
Box plot illustrating the comparison between female and male participants. (°) denote outliers, while (*) indicate extreme values.

A statistically significant relationship was found between the responses of females and males regarding the quality of the presented image using HoloLens 2 goggles (Table 2, Figure 3).

No significant correlation was found between participants with and without prior experience using VR/MR/AR (Table 3).

No significant correlation was found between age and responses (Table 4).

## 4. Discussion

The education process is continually being modified and enriched with new technologies to enhance teaching effectiveness. However, the introduction of a new technology does not automatically ensure its acceptance or improvement of the learning process. At times, such technologies may be difficult to operate or inconvenient to use, which prevents their adoption as routine teaching tools. Nevertheless, demonstrating a new technology and gathering user feedback should constitute the first step toward its broader implementation. Our study sought to investigate how dental students evaluate three-dimensional visualizations of impacted teeth. By collecting and analyzing their opinions, we aimed to provide reliable data on the acceptance and potential benefits of integrating 3D visualization technology into medical university education.

Our findings suggest that students perceive 3D visualization as a valuable enhancement to their learning experience, particularly in understanding the procedures involved in exposing and extracting impacted teeth. These results are consistent with those of Kumar et al. (2021) [1], who reported positive feedback on a similar system validated by 12 experts (plastic surgeons and dermatologists). Their study highlighted the educational value of clearly labeled facial structures with interactive tags, which contributed to better understanding of facial anatomy.

In our study, HoloLens2 mixed-reality goggles were employed. However, this is not the only available tool for immersive 3D visualization. For instance, a similar initiative in France utilized the DIVA system (Data Integration and Visualization in Augmented and Virtual Environments, Pasteur Institute, Paris) to assess user satisfaction. In that study, 92% of undergraduate medical students reported high satisfaction levels when using DIVA to explore craniofacial trauma cases. Although DIVA was also described as intuitive (88%) and well-tolerated (86%), these usability scores were slightly lower than those reported in our study [4].

It is noteworthy that participants largely evaluated the image quality and the ease of image manipulation positively, with only a minority reporting any difficulties. Importantly, previous familiarity with 3D visualization technologies did not have a significant influence on the responses, suggesting that the tool can be successfully applied even among individuals with no prior exposure to such solutions. This aspect is particularly relevant in the context of academic education, where students often encounter new technologies for the first time during their training. It is important to note that differences in experience with visual and gaming technologies may also contribute to variations in perception. The result revealed a statistically significant difference between female and male participants in their assessment of image quality using HoloLens 2 goggles. This may result from the generally lower amount of time that women spend playing video games compared to men [12]. Reduced gaming experience could lead to lower sensitivity in image perception, and thus comparatively lower expectations regarding image quality.

Advanced 3D imaging techniques such as VR, AR, and MR extend beyond education and are now integrated into clinical practice. They are increasingly used for preoperative and intraoperative surgical guidance in orthognathic surgery [3,13], for developing detailed surgical plans [14,15], and for enhancing the acquisition of surgical skills [5]. Familiarity with immersive technologies during undergraduate training may foster a smoother transition into their clinical use and help mitigate existing technical and practical challenges.

This study has several strengths and limitations. A major strength is the inclusion of 82% of all 5th-year dental students, resulting in a relatively homogeneous yet representative sample in terms of age and gender (predominantly female). However, the study is based solely on students’ perceptions, and the collected information reflects only subjective opinions. No objective assessment of actual learning benefits was performed. An additional limitation is the lack of validation of the questionnaire. Consequently, the findings relate to students’ acceptance of the technology and its perceived potential advantages, rather than demonstrated effectiveness. To more accurately verify the educational benefits of three-dimensional visualization with goggles, future research should compare the performance of students using goggles with those receiving traditional instruction alone.

Another limitation, noted by Kumar et al. (2021) [1], is that translucent holograms can reduce the clarity of textures in 3D models, which may affect the visual realism and interpretation of anatomical structures. Finally, although students received instructions on using the HoloLens2, limited prior experience with the device impacted their ability to manipulate the images comfortably. Enhanced user familiarity and prolonged exposure could improve the ease of interaction, which was rated lowest among evaluated aspects.

## 5. Conclusions

The present study highlights the benefits of using 3D visualizations of impacted teeth for fifth-year dental students. The application of mixed-reality (MR) technology appears to facilitate the evaluation of tooth position and enhances understanding of the surgical procedures required for managing impacted teeth. Students responded positively to the integration of 3D visualizations as a supplementary teaching tool, indicating that it improved their comprehension of the subject matter.

Despite these encouraging findings, the quality and manipulability of the visualized images require further improvement. Continued exploration and integration of emerging technologies in the academic setting may contribute to more effective learning methods. Future studies should address the identified limitations to further validate and optimize the use of 3D visualization in dental education.

Nevertheless, the study relies solely on students’ perceptions, and the findings therefore reflect subjective opinions rather than objectively measured learning outcomes. No objective assessment was performed to confirm actual educational benefits. To establish the true effectiveness of MR technology in dental education, future research should incorporate objective outcome measures and direct comparisons with traditional teaching methods.

## Figures and Tables

**Table 1:** Descriptive statistics of responses to questions.

The Three-Dimensional Image of the Impacted Tooth	Median	25th Percentile	75th Percentile	Min	Max
The three-dimensional visualization helped you evaluate the position of the impacted tooth.	1.0	1.0	2.0	1	4
2. You did not have any problems with the image manipulation using the HoloLens 2 goggles.	2.0	2.0	3.0	1	5
3. The quality of the presented image using HoloLens 2 goggles is satisfactory.	2.0	1.0	2.0	1	5
4. Would you like three-dimensional image visualization to be an additional element of teaching classes?	1.0	1.0	2.0	1	4
5. …will benefit you while learning.	1.0	1.0	2.0	1	4
6. …allowed you to better understand the course of the procedure of exposure/extraction of the impacted tooth.	1.0	1.0	2.0	1	4
7. …allowed you to better understand the possible complications associated with the treatment of impacted teeth.	1.5	1.0	2.0	1	4

*n* = 114; 1—I strongly agree, 2—I rather agree, 3—I have no opinion, 4—I rather disagree, 5—I strongly disagree.

**Table 2:** Comparison between the female and the male.

Question	Male (*n* = 29) (Median; Q1, Q3)	Female (*n* = 85) (Median; Q1, Q3)	*p*-Value
1	1.0; Q1 = 1.0, Q3 = 2.0	1.0; Q1 = 1.0, Q3 = 2.0	0.972
2	2.0; Q1 = 1.0, Q3 = 2.5	2.0; Q1 = 2.0, Q3 = 4.0	0.152
3	2.0; Q1 = 2.0, Q3 = 2.5	2.0; Q1 = 1.0, Q3 = 2.0	**0.015**
4	1.0; Q1 = 1.0, Q3 = 2.0	1.0; Q1 = 1.0, Q3 = 1.5	0.268
5	2.0; Q1 = 1.0, Q3 = 2.0	1.0; Q1 = 1.0, Q3 = 2.0	0.886
6	1.0; Q1 = 1.0, Q3 = 2.0	2.0; Q1 = 1.0, Q3 = 2.0	0.631
7	1.0; Q1 = 1.0, Q3 = 2.0	2.0; Q1 = 1.0, Q3 = 2.0	0.923

**Table 3:** Comparison between participants with and without prior experience using VR/MR/AR.

Question (Median; Q1, Q3)	Participants Without Prior Experience (*n* = 15) (Median; Q1, Q3)	Participants with Prior Experience (*n* = 85) (Median; Q1, Q3)	*p*-Value
1	1.0; Q1 = 1.0 Q3 = 1.0	1.0; Q1 = 1.0 Q3 = 2.0	0.061
2	2.0; Q1 = 1.0, Q3 = 2.0	2.0; Q1 = 2.0 Q3 = 3.25	0.353
3	2.0; Q1 = 1.0, Q3 = 2.0	2.0; Q1 = 1.0 Q3 = 2.0	0.963
4	1.0; Q1 = 1.0, Q3 = 1.0	1.0; Q1 = 1.0 Q3 = 2.0	0.540
5	1.0; Q1 = 1.0, Q3 = 2.0	1.5; Q1 = 1.0 Q3 = 2.0	0.134
6	2.0; Q1 = 1.0, Q3 = 2.0	1.0; Q1 = 1.0 Q3 = 2.0	0.737
7	2.0; Q1 = 1.0, Q3 = 2.0	1.0; Q1 = 1.0 Q3 = 2.0	0.933

**Table 4:** Comparison of participants’ ages and responses.

Questionnaire Item	Spearman’s Rank Correlation Coefficient	Age
better understand the possible complications	−0.099	0.295
understand the course of the procedure	−0.085	0.371
benefit you while learning	−0.070	0.456
additional element of teaching classes	0.018	0.853
quality of the presented image	−0.071	0.455
the image manipulation	−0.045	0.635
evaluate the position	0.024	0.799

*n* = 114.

## Data Availability

The data presented in this study are available on request from the corresponding author due to the anonymous nature of the survey responses.
